# Impact of pre-treatment food on praziquantel absorption, metabolism, treatment-associated side effects, and drug efficacy: a systematic review and meta-analysis protocol

**DOI:** 10.1186/s13643-026-03082-4

**Published:** 2026-02-16

**Authors:** Farah Hamdan, Poppy H. L. Lamberton, Lydia Trippler

**Affiliations:** Department for Biodiversity, One Health, and Veterinary Medicine, University of Glasgow, Glasgow, Graham Kerr Building, Science Way, Glasgow, Scotland G12 8QQ UK

**Keywords:** Absorption, Efficacy, Food, Mass drug administration, Metabolism, Pharmacokinetics, Praziquantel, PZQ, Schistosomiasis, Side effects

## Abstract

**Background:**

Praziquantel is the drug of choice for parasitic diseases such as opisthorchiasis, schistosomiasis, and taeniasis. The U.S. Food and Drug Administration and World Health Organization (WHO) advise administering praziquantel with food to increase absorption and decrease side effects. However, there is a scarcity of evidence on the impact of food, and different types of food, on treatment outcomes. This review aims to address this knowledge gap by (i) examining how pre-treatment food intake is reported in humans, and characterising the types of food provided, if any, and (ii) assessing whether the presence and/or type of food impacts four key treatment outcomes in humans: absorption, metabolism, treatment-associated side effects, and drug efficacy.

**Methods:**

We will search the following databases: Embase (via Ovid), MEDLINE (via PubMed), Web of Science Core Collection, Cochrane Central Register of Controlled Trials (CENTRAL), and African Index Medicus (via Global Index Medicus), and trial registries including clinicaltrials.gov and the WHO International Clinical Trials Registry Platform (ICTRP). Additionally, we will perform citation and grey literature searching and will contact researchers to obtain information on ongoing and unpublished relevant research. The search strategy is constructed using two core concepts: (i) praziquantel, and (ii) the outcomes of interest (pharmacokinetics, treatment-associated side effects, and drug efficacy). In Covidence, two review authors will independently screen the retrieved studies based on pre-defined inclusion/exclusion criteria and extract data using a standardised form. Risk of bias will be assessed using the Risk of Bias 2 (RoB 2) tool for randomised controlled trials and the Risk of Bias In Non-randomised Studies of Interventions (ROBINS-I) tool for non-randomised studies. Data will be synthesised narratively, and random-effects meta-analyses will be conducted to estimate the effect of food on the outcomes of interest, where appropriate. The certainty of evidence will be assessed using the Grading of Recommendations Assessment, Development and Evaluation (GRADE) framework.

**Discussion:**

This review has the potential to enhance treatment outcomes for individuals and communities living in endemic regions for schistosomiasis and other parasitic diseases treated with praziquantel. Our findings will be of direct relevance for WHO guidelines and, therefore, can help improve the design and impact of future targeted treatment and mass drug administration (MDA) programmes.

**Systematic review registration:**

PROSPERO CRD420251024296

**Supplementary Information:**

The online version contains supplementary material available at 10.1186/s13643-026-03082-4.

## Background

Praziquantel is a widely used anthelmintic drug, and the drug of choice for the treatment, control, and elimination efforts for several parasitic diseases, especially the neglected tropical diseases (NTDs) opisthorchiasis, schistosomiasis, and taeniasis [1]. Praziquantel is widely used in low- and middle-income countries, where mass drug administration (MDA) programmes are the cornerstone for controlling morbidity, eliminating diseases as a public health problem, and eventually interrupting their transmission [1, 2]. Despite its extensive use, persistent hotspots, treatment failures, and variability in treatment outcomes, such as drug absorption, metabolism, treatment-associated side effects, and drug efficacy, have been observed across settings and populations [3–9]. One potential contributor to this variability is food intake before or at the time of drug administration. Praziquantel exhibits low aqueous solubility and variable bioavailability [4, 10, 11], and evidence from a pharmacokinetic study suggests that food, especially high-carbohydrate meals, greatly enhances drug absorption in comparison to fasting conditions [12].

The U.S. Food and Drug Administration recommends that praziquantel treatment be administered with meals, and since 1993, the World Health Organization (WHO) has also advised administering praziquantel with food to decrease side effects when treating schistosomiasis [11, 13]. In 2022, for the first time, the guidelines for MDA against schistosomiasis also included specific food suggestions, recommending that praziquantel should be administered alongside items such as bread, biscuits, juices, or porridge to increase acceptability and decrease side effects [14]. Furthermore, it is likely that extra food provision only occurs when MDA is implemented alongside school-feeding programmes rather than as a standard component. Given the potential impact of food on the drug bioavailability and side effects, the absence of food provision may lead to a reduction in the intended impact of MDAs. However, research studies might not pick up on this reduced impact as they provide praziquantel with food, resulting in a potential mismatch between observed treatment effects in research studies and in-field practice where MDAs might be conducted without food provision. As the WHO aims to eliminate schistosomiasis as a public health problem globally by the year 2030 [1], a strong evidence base for how praziquantel should be administered is more important than ever. Subsequently, the findings of this systematic review will be timely to help support these elimination efforts.

Despite the potential significance of food intake as a modifying factor, there has been no systematic synthesis of how pre-treatment food consumption is reported in human studies of praziquantel or MDA programmes, nor how different types of food might impact the praziquantel absorption or metabolism, treatment-related side effects, or drug efficacy across the range of parasitic diseases that praziquantel targets. This review aims to address these knowledge gaps by (i) examining how pre-treatment food intake is reported in humans, and if so, characterising the types of food provided, and (ii) assessing whether the presence and/or type of food impacts four key treatment outcomes in humans: absorption, metabolism, treatment-associated side effects, and drug efficacy.

## Methods

### Registration

This protocol was prospectively registered with PROSPERO (CRD420251024296) on the 23rd of June 2025 and has been reported using the Preferred Reporting Items for Systematic Reviews and Meta-Analyses Protocol (PRISMA-P) guidelines (Additional file 1) [15]. The systematic review and meta-analysis will be prepared following standard systematic review methods [16] and reported following the PRISMA 2020 guidelines [17].

### Eligibility criteria

The eligibility criteria for including articles in the review are based on items of the PICO framework [18], complemented by criteria regarding the study design (Table 1).

**Table 1:** Eligibility criteria for publications included in the review

	Domain	Criteria	Application
1	Population	Inclusion: The study is focused on humans, regardless of health status	Title/abstract and full-text review
		Exclusion: The study was conducted in animals only, or was explicitly an *in-vivo*, *in-vitro*, or *in-silico* study	
2	Intervention	Inclusion: Praziquantel was administered to the study participants	Title/abstract and full-text review
		Exclusion: • Praziquantel was not administered to the study participants • Praziquantel was administered simultaneously with, or shortly after, another intervention intended to treat the same condition	
3	Outcome	Inclusion: • Praziquantel absorption, metabolism, treatment-associated side effects, or efficacy were assessed • Efficacy of praziquantel on the infectious agent was assessed using parasitological parameters	Title/abstract and full-text review
		Exclusion: • Praziquantel absorption, metabolism, treatment-associated side effects, or efficacy were not assessed • Information about food consumption is highly unlikely to be retrieved	
4	Publication type	Inclusion: The study is a prospective or retrospective observational study (e.g. cross-sectional, cohort, case-control), or a randomised controlled trial	Title/abstract and full-text review
		Exclusion: • The study is a book chapter, society guideline, review, case report, letter, comment, editorial, conference abstract, or study protocol • The full text of the study is not available	

### Outcomes

The review will focus on three key outcomes related to the impact of pre-treatment food intake on praziquantel treatment in humans:

1. Pharmacokinetic outcomes will be defined as the total drug exposure over time, measured by the area under the curve (AUC) and the maximum plasma concentration reached after drug administration (*C*_max_) for the two drug isomers and main metabolites.
2. Treatment-associated side effects will be defined as adverse events occurring after treatment with praziquantel, such as vomiting, abdominal pain, bloody diarrhoea, or other symptoms reported as treatment-related.
3. Drug efficacy will be defined as worm burden reduction, egg reduction rate, or cure rate after praziquantel treatment, and may also include parasite DNA detection, antigen clearance, cyst resolution, or worm expulsion for the main infections treated with praziquantel.

In both the systematic review and the systematic review protocol, food is defined as including both solid meals and beverages, following the WHO guidelines [14].

### Search strategy

**Fig. 1:**
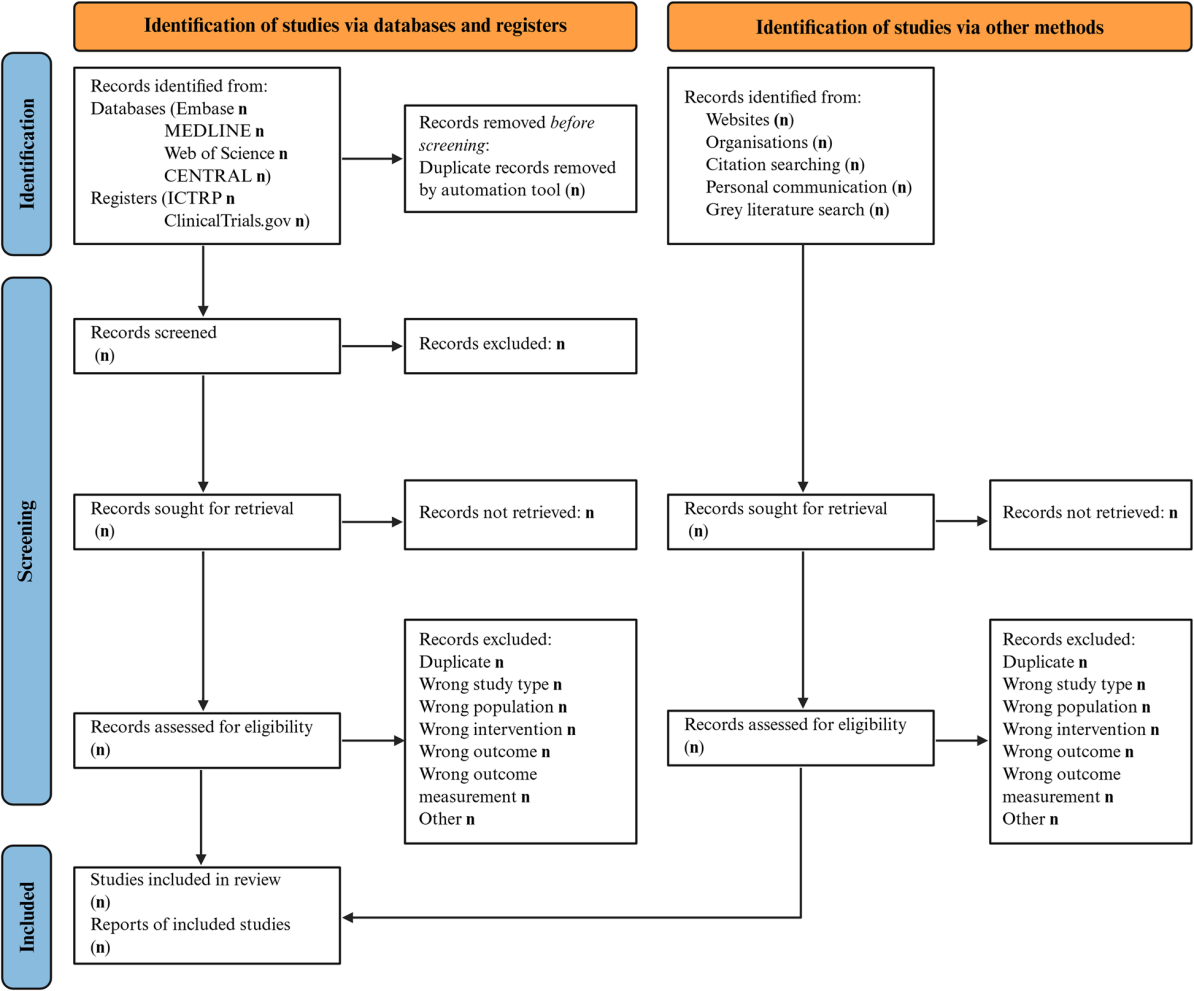
PRISMA flow diagram for article inclusion in the systematic review. The figure was created in BioRender, and licensed under CC-BY [19], and based on the PRISMA guidelines, which are licensed under CC-BY-4.0 [17]

We will conduct a systematic literature search in the databases Embase (via Ovid), MEDLINE (via PubMed), Science Citation Index Expanded (SCI-EXPANDED), Conference Proceedings Citation Index—Science (CPCI-S), Emerging Sources Citation Index (ESCI) via Web of Science, Cochrane Central Register of Controlled Trials (CENTRAL), and African Index Medicus (via Global Index Medicus), as well as in the registries clinicaltrials.gov and the WHO International Clinical Trials Registry Platform (ICTRP) (Fig. 1). In addition to searching electronic databases, we will inspect the reference list of relevant studies and locate any other articles that were cited to retrieve additional important records that might have been missed during the database search. We will also contact key experts to inquire about ongoing or unpublished related studies. In addition, grey literature repositories, as well as subject-specific and other relevant websites, will be searched.

The search strategy will be constructed using both controlled vocabulary, when available in a given database, and text words, combined using Boolean operators, and will be based on two key concepts: (i) praziquantel; and (ii) the three outcomes of interest: pharmacokinetics, side effects, and drug efficacy. The side effects search block (line #3 in Table 2) was adapted from a validated search strategy [20] and enhanced with side effects terms related to praziquantel. The concepts were further refined by applying publication type restrictions and excluding animal-only studies. The detailed search strategy for Embase is presented in Table 2. The search strategies for the other databases and registers are provided in Additional file 2.

**Table 2:** Search term for Embase

#1	exp praziquantel/or (praziquantel or PZQ or Biltricide).ab,kw,ti
#2	exp pharmacokinetics/OR exp bioavailability/OR (pharmacokinetic* OR "drug kinetics" OR liberation OR absorption OR clearance OR “absorption rate constant” OR K_a_ OR metabolism OR bioavailability OR "drug plasma level*" OR “plasma concentration*” OR LADMER OR ADMET OR ADME OR ADME-Tox OR “maximum plasma concentration” OR “maximum serum concentration” OR Cmax OR C-max OR "area under the curve" OR AUC OR half-life OR 4-OH-PZQ OR R-PZQ OR S-PZQ OR 4-OH-PZQ OR R-trans-4-OH-PZQ OR R-cis-4-OH-PZQ).ab,kw,ti
#3	Ae.fs OR Safe*.ti,ab,kw OR Adverse.ti,ab,kw,ox OR Po.fs OR Co.fs OR exp adverse drug reaction/OR Complication*.ti,ab OR Drug safety/OR To.fs OR Side effect*.ti,ab OR Risk.ti OR Tolerance.ti,ab OR Tolerated.ti,ab OR Harm.ti,ab OR Side reaction*.ti,ab OR drug withdrawal/OR health risks.ti,ab OR potential risks.ti,ab OR toxic effects.ti,ab OR toxicity.ti,ab OR toxicities.ti,ab OR headache.ab,kw,ti OR dizziness.ab,kw,ti OR gastrointestinal.ab,kw,ti OR abdominal.ab,kw,ti OR nausea.ab,kw,ti OR vomiting.ab,kw,ti OR fatigue.ab,kw,ti OR urticaria.ab,kw,ti OR diarrh?ea.ab,kw,ti OR fever.ab,kw,ti OR pyrexia.ab,kw,ti OR malaise.ab,kw,ti
#4	exp treatment outcome/OR exp drug efficacy/OR exp antiparasitic activity/OR (“treatment outcome” OR “treatment effic*” OR “clinical effic*” OR “drug potency” OR “drug effic*” OR “therapeutic effect” OR “clinical outcome” OR “health outcome” OR “patient outcome” OR “intervention outcome” OR “treatment response” OR “drug response” OR “therapeutic response” OR “dose response” OR “treatment effectiveness” OR “biological response” OR ERR OR “egg* reduction” OR “worm* reduction” OR “worm* burden reduction” OR CR OR “cure rate” OR “praziquantel effic*” OR “PZQ effic*” OR cyst OR cysts OR MRI OR “magnetic resonance imaging” OR calcification* OR sputum OR “prevalence reduction” OR CCA OR CAA OR “circulating cathodic antigen*” OR “circulating anodic antigen*” OR “geometric mean intensity” OR GMI OR “worm expulsion” OR "Kato Katz" OR "Kato-Katz" OR efficacy OR “G score” OR “G-score” OR “urine filtration” OR “microscopy”).ab,kw,ti
#5	Comment/or Letter/or Editorial/or exp Review/
#6	animal/
#7	human/
#8	6 not (6 and 7)
#9	2 or 3 or 4
#10	1 and 9
#11	10 not (5 or 8)

### Data management, selection process, and data collection process

All articles will be extracted from the databases and registries and imported into Covidence, where duplicates will be identified and removed [21]. Using Covidence, two review authors will independently screen titles and abstracts for the eligibility criteria of population, intervention, outcome, and study type (Table 1). Discrepancies between the two review authors will be resolved through discussion, and if needed, a third reviewer will mediate to reach a consensus. In a second step, two independent review authors will continue with full-text screening of the included articles and decide based on the criteria of population, intervention, outcome, and study type about their eligibility for inclusion in the review. Again, discrepancies between the two review authors will be resolved through discussion, and if needed, a third reviewer will mediate to reach a consensus. Publications in a language other than English will be translated using an artificial intelligence tool (ChatGPT, Open AI, 2025).

### Data extraction

From each study included in the analysis, we will extract information about the study design, year of publication, year of study, number of participants, study location, information about the study population’s demographics (e.g. sex, age, socioeconomic status, individual or community praziquantel treatment history, community endemicity), health status at recruitment (i.e. healthy population-based study or diseased/infected patient-based study), infection and co-infection data, information about the administered praziquantel drug (e.g. brand, dosage, co-administration with other drugs, treatment plan), information related to drug efficacy measurements (e.g. diagnostic test used, number of specimens tested, time intervals of praziquantel efficacy measurement post treatment), and information on the feeding status (e.g. food given/not given/fasted/not mentioned, if food was given then the type of food/not described). Furthermore, we will extract data to perform the meta-analyses, including but not limited to odds ratios (ORs) with confidence intervals (CIs), drug efficacy measurements, the number of participants who did or did not experience the outcome of interest (e.g. side effects), and the mean of *C*_max_ and AUC in pharmacokinetic studies.

A data extraction form will be piloted, and two review authors will independently collect the data using Covidence, and any discrepancies will be resolved through discussion, and if needed, a third reviewer will mediate to reach a consensus.

If important data for calculating effect measures or assessing the risk of bias is missing or unclear, the corresponding author’s of each study will be contacted by email. A reminder will be sent within two weeks in case of no response, and we will wait two more weeks before progressing without the relevant information.

### Risk of bias in individual studies

We will assess the risk of bias for each individual study at the outcome level for the outcome(s) of interest that the study examined (i.e. praziquantel pharmacokinetics, treatment-associated side effects, and/or drug efficacy). We will use two Cochrane tools: (i) the Risk of Bias 2 (RoB 2) tool to assess the risk of bias in randomised controlled studies; and (ii) the Risk of Bias in Non-randomised Studies of Interventions (ROBINS-I) tool to assess the risk of bias in non-randomised studies [22, 23].

Five fixed sets of domains will be assessed using the RoB 2 tool; these are

1. Bias arising from the randomisation process
2. Bias due to deviations from intended interventions
3. Bias due to missing outcome data
4. Bias in measurement of the outcome
5. Bias in selection of the reported result

And seven sets of domains will be assessed using ROBINS-I; these are

1. Risk of bias due to confounding
2. Risk of bias in classification of interventions
3. Risk of bias in selection of participants into the study (or into the analysis)
4. Risk of bias due to deviations from intended interventions
5. Risk of bias due to missing data
6. Risk of bias arising from measurement of the outcome
7. Risk of bias in selection of the reported result

To arrive at a final judgment of the risk of bias of an outcome using both ROB 2 and ROBINS-I tools, we will map the individual risk of bias judgments for each assessed domain to an overall risk of bias for that outcome following recommendations. The overall risk of bias for the assessed outcome will be either “low”, “some concerns”, or “high” using RoB2 and either “low”, “moderate”, “serious”, “critical”, or “no information” using ROBINS-I.

Two review authors will independently conduct the quality assessment. Discrepancies between the two assessors will be resolved through discussion, and if needed, a third reviewer will mediate to reach a consensus. If all studies to be included in the same meta-analysis are at the same risk of bias, they will all be used to estimate the intervention effect, with a description of the risk of bias for each domain. If the studies to be pooled in a meta-analysis are not at the same risk of bias, we will conduct a stratified analysis based on the overall risk of bias.

### Statistical analysis

We will conduct a descriptive analysis of the included studies, focusing on the outcome assessed and whether the fed or fasting state prior to praziquantel treatment was reported. Preliminary, narrative synthesis of the included studies will be performed to examine their findings on the effect of food provision prior to praziquantel treatment on the outcomes of interest (pharmacokinetics, treatment-associated side effects, or drug efficacy). Additionally, we will assess within and between studies relationships to define factors that can explain any potential variation in the intervention’s effect size and/or direction. Finally, an assessment of the evidence strength will be carried out before drawing our conclusions. If food provision was reported, we will provide a narrative synthesis describing the type of food individuals consumed or the duration of fasting prior to praziquantel administration.

In addition to the narrative synthesis, we will conduct two separate meta-analyses. The first meta-analysis will include studies that directly compared fed *versus* fasting individuals (i.e. within-study comparisons) and, if possible, the type of food given. The second meta-analysis will include studies that did not compare fed and fasted individuals within the same study, but in which all participants were either fed or non-fed/fasted (i.e. between-study comparisons) and, if possible, between the different types of food given.

For all meta-analyses, studies will be eligible if they assessed the same outcome (e.g. praziquantel pharmacokinetics, treatment-associated side effects, or drug efficacy) using the same or comparable measures, and they provided complete effect estimates or sufficient information to compute them, either in the publication or through author contact. All analyses will be random-effects meta-analyses, using the inverse-variance method with 90% CIs for pharmacokinetics estimates, as typically done for pharmacokinetics studies [12, 24], and 95% CIs for side effects and drug efficacy estimates, to estimate the intervention effect, and the results will be shown in forest plots.

Log ORs will be used as an effect measure to compare dichotomous data for the side effects outcome. ORs will be extracted from studies that report on them or will be calculated from studies that did not and will be pooled with a 95% CI. For continuous data of the pharmacokinetics and drug efficacy outcomes, the mean difference (MD) will be calculated as the effect measure where the same scale is used to measure the outcome of interest, and the standardised mean difference (SMD) will be used as the effect measure where the outcome was measured on different scales; both MD and SMD will be reported with a 95% CI and with a 90% CI for the pharmacokinetic outcome.

Heterogeneity will be assessed following the meta-analysis by visual inspection, a *χ*^2^ test, and the *I*^2^ statistic. If heterogeneity is detected, a meta-regression subgroup analysis will be carried out on subgroups depending on the papers included, for example:

- Endemic *versus* non-endemic areas
- Non-infected/non-detected infections *versus* infected/diseased participants
- Adults *versus* children and adolescents
- Different praziquantel dosage/treatment regimen

We will undertake a sensitivity analysis, if feasible, to assess the effect of some decisions we made on the results of the meta-analysis:

- The choice of summary statistics used as effect measures (e.g. what impact the choice of OR over risk ratio (RR) might have on the results of the meta-analysis)
- Fixed-effect meta-analysis

### Meta-biases

Funnel plots will be visually inspected to assess the risk of publication bias. Additionally, we will conduct tests (e.g. the Egger’s regression test or the Begg’s rank correlation test) to evaluate the symmetry of the funnel plot, and a *p*-value of less than 0.1 will be interpreted as evidence of a publication bias. Further, to detect non-reporting bias, we will compare the outcomes reported in the published articles to available documents detailing the outcomes of interest from before the start of the study (e.g. published protocols and trial registries).

### Confidence in cumulative evidence

To assess the certainty of the body of evidence, we will use the Grading of Recommendations Assessment, Development, and Evaluation (GRADE) approach [25].

For randomised trials, we will evaluate the following five domains:

1. Limitations in study design or execution (risk of bias)
2. Inconsistency of results
3. Indirectness of evidence
4. Imprecision
5. Publication bias

For non-randomised studies, three additional domains will be considered:

1. Large magnitude of effect
2. Plausible confounding
3. Dose–response gradient

In line with GRADE recommendations, we will begin with a “high” certainty rating for randomised trials and with a “low” certainty rating for non-randomised studies. The certainty will then be downgraded by one or more levels when concerns are identified in the five core domains. For non-randomised studies, the three additional factors will be considered for potential upgrading of certainty, if warranted.

For each outcome of interest assessed, the certainty of evidence will be rated as “high”, “moderate”, “low”, or “very low”. Two study authors will independently conduct the assessments, resolve any disagreements through discussion, and clearly document all judgments leading to the final grading.

The results will be displayed in a summary of findings table, which will be created using the GRADE Working Group’s software, GRADEpro GDT (www.gradepro.org).

## Discussion

Praziquantel is a widely used anthelmintic drug for the treatment of several parasitic infections, including opisthorchiasis, schistosomiasis, and taeniasis. Each year, hundreds of millions of praziquantel tablets are distributed globally, primarily through MDA campaigns targeting schistosomiasis control and elimination [2]. Despite longstanding recommendations to administer praziquantel with food, and although there is evidence to suggest that food can impact praziquantel-related outcomes, such as absorption, metabolism, treatment-associated side effects, and drug efficacy [12, 26], many control programmes do not administer praziquantel with food. In general, there is a lack of systematically synthesised evidence on whether, and how, food impacts treatment outcomes and how this may impact the short-term and long-term success of treatment programmes. This review will address this gap and help inform both clinical guidance and implementation strategies in targeted treatment and MDA programmes.

One potential limitation of this review is the anticipated variation in study designs, populations, and the measurements of the different outcomes, which may limit the ability to draw definitive conclusions. In addition, the feeding or fasting status of the participants may be poorly reported. To address the potential limitations, we will include a narrative synthesis and contact study authors to attempt to capture the complete extent of food-related information.

Despite these challenges, this systematic review is expected to make an important contribution to treatment guidelines. It will be the first to examine how food co-administration with praziquantel is reported in humans, to characterise the food types provided, and to evaluate the existing evidence on the impact of pre-treatment food intake on the drug’s absorption, metabolism, treatment-associated side effects, and drug efficacy. The findings may inform future clinical trials, improve the design and implementation of MDA programmes, and inform WHO recommendations, decision makers, researchers, and practitioners on best practices. This review has the potential to enhance treatment outcomes for individuals and communities living in endemic regions for schistosomiasis and other parasitic infections treated with praziquantel through an improved evidence base that can help inform policy.

## Supplementary Information

Additional file 1: PRISMA-P checklist for information reported in the protocol.

Additional file 2: Search strategies for the different databases and registries used in the study.

## Data Availability

Not applicable.

## Abbreviations

AUC: Area under the curve
CI: Confidence interval
*C*_max_: Maximum plasma concentration
ERR: Egg reduction rate
GRADE: Grading of Recommendations Assessment, Development and Evaluation
MDA: Mass drug administration
OR: Odds ratio
PRISMA: Preferred Reporting Items for Systematic reviews and Meta-Analyses
PRISMA-P: Preferred Reporting Items for Systematic reviews and Meta-Analyses Protocol
ROB 2: Risk Of Bias 2
ROBINS–I: Risk Of Bias In Non-randomised Studies of Interventions
WHO: World Health Organization
