# Supplementary material for: Impact of pre-treatment food on praziquantel absorption, metabolism, treatment-associated side effects, and drug efficacy: a systematic review and meta-analysis protocol
Source: Syst Rev. 2026 Feb 16;15:91. doi: 10.1186/s13643-026-03082-4 (PMC13011758; doi:10.1186/s13643-026-03082-4)
Supplement: Supplementary file 2 — Additional file 2: Search strategies for the different databases and registries used in the study. [file 13643_2026_3082_MOESM2_ESM.pdf]

Additional Table 2: Search strategies for the different databases and registries used in the study.

**MEDLINE via PubMed:**	
#1	"praziquantel" [Mesh] OR praziquantel [tiab] OR PZQ [tiab] OR Biltricide [tiab]
#2	"Pharmacokinetics"[Mesh] OR "Biological Availability"[Mesh] OR pharmacokinetic* [tiab] OR "drug kinetics"[tiab] OR liberation[tiab] OR absorption[tiab] OR clearance[tiab] OR "absorption rate constant"[tiab] OR Ka[tiab] OR metabolism[tiab] OR bioavailability[tiab] OR "drug plasma level*"[tiab] OR "plasma concentration*"[tiab] OR LADMER[tiab] OR ADMET[tiab] OR ADME[tiab] OR ADME-Tox[tiab] OR "maximum plasma concentration"[tiab] OR "maximum serum concentration"[tiab] OR Cmax[tiab] OR C-max[tiab] OR "area under the curve"[tiab] OR AUC[tiab] OR half-life[tiab] OR 4-OH-PZQ[tiab] OR R-PZQ[tiab] OR S-PZQ[tiab] OR 4-OH-PZQ[tiab] OR R-trans-4-OH-PZQ[tiab] OR R-cis-4-OH-PZQ[tiab]
#3	"Adverse Effects"[sh:noexp] OR Safe*[tiab] OR "Drug Effects"[sh:noexp] OR "Adverse"[tiab] OR "Complications"[sh:noexp] OR Side effect*[tiab] OR Complication*[tiab] OR "Chemically Induced"[sh:noexp] OR "Tolerated"[tiab] OR "Tolerance"[tiab] OR Harm*[tiab] OR "Toxicity"[tiab] OR "Risk"[ti] OR "Pregnancy complications/Drug Therapy"[mesh:noexp] OR "Clinical trial, phase IV"[pt] OR "Drug hypersensitivity"[mesh:noexp] OR "Tolerability"[tiab] OR "Toxicity"[sh:noexp] OR "Toxicology"[mesh:noexp] OR "Drug induced"[tiab] OR "Negative effects"[tiab] OR headache[tiab] OR dizziness[tiab] OR gastrointestinal[tiab] OR abdominal[tiab] OR nausea[tiab] OR vomiting[tiab] OR fatigue[tiab] OR urticaria[tiab] OR diarrh*ea[tiab] OR fever[tiab] OR pyrexia[tiab] OR malaise[tiab]
#4	"Treatment Outcome"[Mesh] OR "treatment outcome"[tiab] OR "treatment effic*"[tiab] OR "clinical effic*"[tiab] OR "drug potency"[tiab] OR "drug effic*"[tiab] OR "therapeutic effect"[tiab] OR "clinical outcome"[tiab] OR "health outcome"[tiab] OR "patient outcome"[tiab] OR "intervention outcome"[tiab] OR "treatment response"[tiab] OR "drug response"[tiab] OR "therapeutic response"[tiab] OR "dose response"[tiab] OR "treatment effectiveness"[tiab] OR "biological response"[tiab] OR ERR[tiab] OR "egg reduction"[tiab] OR "eggs reduction"[tiab] OR "worm* reduction"[tiab] OR "worm* burden reduction"[tiab] OR CR[tiab] OR "cure rate"[tiab] OR "praziquantel effic*"[tiab] OR "PZQ effic*"[tiab] OR cyst[tiab] OR cysts[tiab] OR MRI[tiab] OR "magnetic resonance imaging"[tiab] OR calcification*[tiab] OR sputum[tiab] OR "prevalence reduction"[tiab] OR CCA[tiab] OR CAA[tiab] OR "circulating cathodic antigen*"[tiab] OR "circulating anodic antigen*"[tiab] OR "geometric mean intensity"[tiab] OR GMI[tiab] OR "worm expulsion"[tiab] OR "Kato Katz"[tiab] OR "Kato-Katz"[tiab] OR efficacy[tiab] OR "G score" [tiab] OR "G-score" [tiab] OR "urine filtration"[tiab] OR "microscopy"[tiab]
#5	"Comment" [Publication Type] OR "Letter" [Publication Type] OR "Editorial" [Publication Type] OR "Review" [Publication Type]
#6	"Animals"[Mesh:noexp]
#7	"Humans"[Mesh]
#8	#6 not (#6 AND #7)
#9	#2 OR #3 OR #4
#10	#1 AND #9
#11	#10 NOT (#5 OR #8)

**Web of Science Core Collection**<br>**Science Citation index Expanded (SCI-EXPANDED), Conference Proceedings Citation Index - Science (CPCI-S), Emerging Sources Citation Index (ESCI)**	
#1	**TI=(**praziquantel OR PZQ OR Biltricide**)** OR **AB=(**praziquantel OR PZQ OR Biltricide**)** OR **AK=(**praziquantel OR PZQ OR Biltricide**)**
#2	**TI=(**pharmacokinetic* OR "drug kinetics" OR liberation OR absorption OR clearance OR “absorption rate constant” OR Ka OR metabolism OR bioavailability OR "drug plasma level*" OR “plasma concentration*” OR LADMER OR ADMET OR ADME OR ADME-Tox OR “maximum plasma concentration” OR “ maximum serum concentration” OR Cmax OR C-max OR “area under the curve” OR AUC OR half-life OR 4-OH-PZQ OR R-PZQ OR S-PZQ OR 4-OH-PZQ OR R-trans-4-OH-PZQ OR R-cis-4-OH-PZQ**)** OR **AB=(**pharmacokinetic* OR "drug kinetics" OR liberation OR absorption OR clearance OR “absorption rate constant” OR Ka OR metabolism OR bioavailability OR "drug plasma level*" OR “plasma concentration*” OR LADMER OR ADMET OR ADME OR ADME-Tox OR “maximum plasma concentration” OR “ maximum serum concentration” OR Cmax OR C-max OR “area under the curve” OR AUC OR half-life OR 4-OH-PZQ OR R-PZQ OR S-PZQ OR 4-OH-PZQ OR R-trans-4-OH-PZQ OR R-cis-4-OH-PZQ**)** OR **AK=(**pharmacokinetic* OR "drug kinetics" OR liberation OR absorption OR clearance OR “absorption rate constant” OR Ka OR metabolism OR bioavailability OR "drug plasma level*" OR “plasma concentration*” OR LADMER OR ADMET OR ADME OR ADME-Tox OR “maximum plasma concentration” OR “ maximum serum concentration” OR Cmax OR C-max OR “area under the curve” OR AUC OR half-life OR 4-OH-PZQ OR R-PZQ OR S-PZQ OR 4-OH-PZQ OR R-trans-4-OH-PZQ OR R-cis-4-OH-PZQ**)**
#3	**TI=(**Safe* OR Adverse OR "adverse drug reaction" OR Complication* OR "Side effect*" OR Risk OR Tolerance OR Tolerated OR Harm OR "Side reaction*" OR "drug withdrawal" OR "health risks" OR "potential risks" OR "toxic effects" OR toxicity OR toxicities OR headache OR dizziness OR gastrointestinal OR abdominal OR nausea OR vomiting OR fatigue OR urticaria OR diarrh$ea OR fever OR pyrexia OR malaise**)** OR **AB=(** Safe* OR Adverse OR "adverse drug reaction" OR Complication* OR "Side effect*" OR Tolerance OR Tolerated OR Harm OR "Side reaction*" OR "drug withdrawal" OR "health risks" OR "potential risks" OR "toxic effects" OR toxicity OR toxicities OR headache OR dizziness OR gastrointestinal OR abdominal OR nausea OR vomiting OR fatigue OR urticaria OR diarrh$ea OR fever OR pyrexia OR malaise**)** OR **AK=(**Safe* OR Adverse OR "adverse drug reaction" OR "drug withdrawal" OR headache OR dizziness OR gastrointestinal OR abdominal OR nausea OR vomiting OR fatigue OR urticaria OR diarrh$ea OR fever OR pyrexia OR malaise**)**
#4	**TI=(**"treatment outcome" OR "treatment effic*" OR "clinical effic*" OR "drug potency" OR "drug effic*" OR "therapeutic effect" OR "clinical outcome" OR "health outcome" OR "patient outcome" OR "intervention outcome" OR "treatment response" OR "drug response" OR "therapeutic response" OR "dose response" OR "treatment effectiveness" OR "biological response" OR ERR OR "egg* reduction" OR "worm* reduction" OR "worm* burden reduction" OR CR OR “cure rate” OR "praziquantel effic*" OR "PZQ effic*" OR cyst OR cysts OR MRI OR “magnetic resonance imaging” OR calcification* OR sputum OR “prevalence reduction” OR *CCA OR *CAA OR “circulating cathodic antigen*” OR “circulating anodic antigen*” OR “geometric mean intensity” OR GMI OR “worm expulsion” OR "Kato Katz" OR "Kato-Katz" OR efficacy OR “G score” OR “G-score” OR “urine filtration” OR “microscopy”**)** OR **AB=(**"treatment outcome" OR "treatment effic*" OR "clinical effic*" OR "drug potency" OR "drug effic*" OR "therapeutic effect" OR "clinical outcome" OR "health outcome" OR "patient outcome" OR "intervention outcome" OR "treatment response" OR "drug response" OR "therapeutic response" OR "dose response" OR "treatment effectiveness" OR "biological response" OR ERR OR "egg* reduction" OR "worm* reduction" OR "worm* burden reduction" OR CR OR “cure rate” OR "praziquantel effic*" OR "PZQ effic*" OR cyst OR cysts OR MRI OR “magnetic resonance imaging” OR calcification* OR sputum OR “prevalence reduction” OR CCA OR CAA OR “circulating cathodic antigen*” OR “circulating anodic antigen*” OR “geometric mean intensity” OR GMI OR “worm expulsion” OR "Kato Katz" OR "Kato-Katz" OR efficacy OR “G score” OR “G-score” OR “urine filtration” OR “microscopy”**)** OR **AK=(**"treatment outcome" OR "treatment effic*" OR "clinical

	effic*" OR "drug potency" OR "drug effic*" OR "therapeutic effect" OR "clinical outcome" OR "health outcome" OR "patient outcome" OR "intervention outcome" OR "treatment response" OR "drug response" OR "therapeutic response" OR "dose response" OR "treatment effectiveness" OR "biological response" OR ERR OR "egg* reduction" OR "worm* reduction" OR "worm* burden reduction" OR CR OR “cure rate” OR "praziquantel effic*" OR "PZQ effic*" OR cyst OR cysts OR MRI OR “magnetic resonance imaging” OR calcification* OR sputum OR “prevalence reduction” OR CCA OR CAA OR “circulating cathodic antigen*” OR “circulating anodic antigen*” OR “geometric mean intensity” OR GMI OR “worm expulsion” OR "Kato Katz" OR "Kato-Katz" OR efficacy OR “G score” OR “G-score” OR “urine filtration” OR “microscopy”)
#5	"#2 OR #3 OR #4"
#6	"#1 AND #5"
**Cochrane Central Register of Controlled Trials (CENTRAL)**	
#1	MeSH descriptor: [praziquantel] explode all trees
#2	(praziquantel OR PZQ OR Biltricide):ti,ab,kw
#3	MeSH descriptor: [Pharmacokinetics] explode all trees
#4	MeSH descriptor: [Biological Availability] explode all trees
#5	(pharmacokinetic* OR "drug kinetics" OR liberation OR absorption OR clearance OR "absorption rate constant" OR Ka OR metabolism OR bioavailability OR ("drug plasma" NEXT level*) OR (plasma NEXT concentration*) OR LADMER OR ADMET OR ADME OR ADME-Tox OR "maximum plasma concentration" OR "maximum serum concentration" OR Cmax OR "area under the curve" OR AUC OR half-life OR R-PZQ OR S-PZQ OR "R cis 4 OH PZQ" OR "R trans 4 OH PZQ" OR "4 OH PZQ" OR "4 OH PZQ"):ti,ab,kw
#6	MeSH descriptor: [Drug-Related Side Effects and Adverse Reactions] explode all trees
#7	Ae,fs OR Safe*:ti,ab,kw OR Adverse:ti,ab,kw OR Po,fs OR Co,fs OR Complication*:ti,ab OR "Drug safety":ti,ab,kw OR To,fs OR (Side NEXT effect*):ti,ab OR Risk:ti OR Tolerance:ti,ab OR Tolerated:ti,ab OR Harm:ti,ab OR (Side NEXT reaction*):ti,ab OR drug withdrawal:ti,ab,kw OR "health risks":ti,ab OR "potential risks":ti,ab OR "toxic effects":ti,ab OR toxicity:ti,ab OR toxicities:ti,ab OR headache:ab,kw,ti OR dizziness:ab,kw,ti OR gastrointestinal:ab,kw,ti OR abdominal:ab,kw,ti OR nausea:ab,kw,ti OR vomiting:ab,kw,ti OR fatigue:ab,kw,ti OR urticaria:ab,kw,ti OR diarrh?ea:ab,kw,ti OR fever:ab,kw,ti OR pyrexia:ab,kw,ti OR malaise:ab,kw,ti
#8	MeSH descriptor: [Treatment Outcome] explode all trees
#9	("treatment outcome" OR (treatment NEXT effic*) OR (clinical NEXT effic*) OR "drug potency" OR (drug NEXT effic*) OR "therapeutic effect" OR "clinical outcome" OR "health outcome" OR "patient outcome" OR "intervention outcome" OR "treatment response" OR "drug response" OR "therapeutic response" OR "dose response" OR "treatment effectiveness" OR "biological response" OR ERR OR (egg* NEXT reduction) OR (worm* NEXT reduction) OR (worm* NEXT "burden reduction") OR CR OR "cure rate" OR (praziquantel NEXT effic*) OR (PZQ NEXT effic*) OR cyst OR cysts OR MRI OR "magnetic resonance imaging" OR calcification* OR sputum OR "prevalence reduction" OR *CCA OR *CAA OR ("circulating cathodic" NEXT antigen*) OR ("circulating anodic" NEXT antigen*) OR "geometric mean intensity" OR GMI OR "worm expulsion" OR "Kato Katz" OR efficacy OR "G score" OR "urine filtration" OR "microscopy"):ab,kw,ti
#10	#3 OR #4 OR #5 OR #6 OR #7 OR #8 OR #9
#11	#1 OR #2
#12	#10 AND #11

**African Index Medicus via Global Index Medicus**	
#1	**ti:**(mh: D03.633.100.531.690* OR **tw**:(praziquantel OR PZQ OR Biltricide)) OR **ab:**(mh: D03.633.100.531.690* OR tw:(praziquantel OR PZQ OR Biltricide)) OR **kw:**(mh: D03.633.100.531.690* OR tw:(praziquantel OR PZQ OR Biltricide))
**ClinicalTrials.gov**	
#1	praziquantel OR PZQ OR Biltricide
**World Health Organization – International Clinical Trials Registry Platform (ICTRP)**	
#1	praziquantel OR PZQ OR Biltricide
